# Risk Factors and Clinical Outcomes in Nonagenarians with Acute Coronary Syndrome: A Case-Control Study

**DOI:** 10.3390/jcm14051761

**Published:** 2025-03-06

**Authors:** Zeynep Ece Demirbaş, Gönül Zeren, Fatma Can, Can Yücel Karabay

**Affiliations:** 1 Department of Internal Medicine, Dr. Siyami Ersek Thoracic and Cardiovascular Surgery Training and Research Hospital, İstanbul 34668, Turkey; 2 Department of Cardiology, Dr. Siyami Ersek Thoracic and Cardiovascular Surgery Training and Research Hospital, İstanbul 34668, Turkey; gonulzeren@hotmail.com (G.Z.); drftmcan@yahoo.com.tr (F.C.); karabaymd@yahoo.com (C.Y.K.)

**Keywords:** nonagenarians, acute coronary syndrome (ACS), percutaneous coronary intervention (PCI)

## Abstract

**Objective:** With the growing number of individuals over the age of 90 (nonagenarians), understanding the risk factors and clinical outcomes associated with acute coronary syndrome (ACS) in this population has become increasingly important. This study aims to compare demographic, biochemical, and clinical parameters between nonagenarian ACS patients and a control group of healthy individuals within the same age bracket, as well as to analyze differences within the ACS group according to myocardial infarction type and evaluate the impact of percutaneous coronary intervention (PCI) on in-hospital mortality. **Methods:** 104 patients aged 90–100 years diagnosed with ACS for the first time between January 2022 and January 2024 were included in this retrospectively designed case-control study. The patients were categorized into ST-elevation myocardial infarction (STEMI) and non-ST-elevation myocardial infarction (NSTEMI) groups based on their electrocardiographic findings. The control group included 113 healthy individuals within the same age range with no prior history of coronary artery disease. Data on traditional risk factors, including lipid profiles and family history, were analyzed using logistic regression models. Additionally, differences in clinical outcomes, including the length of hospital stay and mortality rates, were evaluated based on the application of PCI. **Results:** The ACS group exhibited significantly higher glucose, white blood cell count, and total cholesterol levels, along with lower hemoglobin and mean corpuscular volume, compared to the control group (*p* < 0.05). While no significant difference was found in low-density lipoprotein (LDL) levels, high-density lipoprotein (HDL) levels were significantly lower in ACS patients (*p* < 0.001). Family history played a more substantial role in the STEMI group compared to the NSTEMI group (*p* = 0.049). Additionally, STEMI patients were more likely to undergo invasive procedures, which were associated with reduced in-hospital mortality (*p* = 0.042). In contrast, no significant difference in mortality was observed in the NSTEMI group based on PCI status. **Conclusions:** This study highlights the distinct risk profiles of elderly ACS patients, emphasizing the critical role of low HDL levels and family history, particularly in STEMI cases. Furthermore, PCI was shown to reduce in-hospital mortality rates in STEMI patients, suggesting that invasive treatment approaches may be beneficial even in this vulnerable population. Personalized and multidisciplinary management strategies are essential for this vulnerable population. Further prospective research is needed to validate these findings and guide clinical decision-making for nonagenarians.

## 1. Introduction

The global aging trend has resulted in a substantial rise in the population aged 90 and above. As of 2024, there were approximately 23 million nonagenarians worldwide, with projections indicating this number will triple by 2050 [1]. This demographic shift increases the healthcare demands of elderly people, particularly in cardiovascular disease management.

Coronary artery disease (CAD) is recognized as the foremost cause of mortality among nonagenarians, with a cardiovascular disease prevalence of 24.1% and a CAD prevalence of 10.9% [2,3,4]. Nonagenarians present a different risk profile for acute coronary syndrome (ACS) development compared to younger populations. Age-related atherosclerosis, endothelial dysfunction, arterial calcification, and multiple comorbidities are the main factors that trigger the development of CAD in this group [5]. Furthermore, risk factors such as hypertension (HT), diabetes mellitus (DM), and chronic renal failure are more prevalent in the older age group, and many of these patients struggle with additional age-related health problems such as frailty, cognitive impairments, and functional decline [2,6]. Compared to patients under 70 years of age with ACS, all-cause and cardiovascular mortality is 20 times higher in this group [7].

Invasive treatment approaches for ACS in nonagenarians require careful consideration due to multiple comorbidities and frailty. However, percutaneous coronary intervention (PCI) stands out as an effective method to reduce mortality in this age group [8]. Studies have shown that percutaneous coronary intervention (PCI) significantly reduces mortality in this age group, with one-year mortality rates of 46% in medically treated patients compared to 24% in those receiving PCI [9]. In light of this information, the rates of invasive intervention in the elderly patient group have been increasing rapidly in recent years [10].

A more personalized, multidisciplinary approach is needed to address the unique needs of nonagenarians with ACS. These challenges, which increase with age, necessitate more clinical research for the older age group and require updating treatment guidelines for the special needs of this group [11]. This study aims to provide a basis for both preventive health services in terms of cardiovascular disease risks during the healthy aging process and customized treatment approaches for this age group after ACS by revealing the differences between healthy controls of the same age group and patients diagnosed with ACS.

## 2. Materials and Methods

This retrospectively designed case-control study included patients aged 90–100 years who were admitted to our hospital’s emergency department between January 2022 and January 2024 with chest pain and were diagnosed with ACS for the first time. ACS diagnosis was based on electrocardiographic findings and/or elevated cardiac enzymes accompanying typical chest pain within the last 12 h. In total, 29 of 104 patients (27.9%) had persistent ST segment elevation on two consecutive ECGs and were included in the ST-elevation myocardial infarction (STEMI) group; the other 75 patients (72.1%) had ECG findings other than ST segment elevation and were included in the non-ST-elevation myocardial infarction (NSTEMI) group.

The control group consisted of 113 healthy individuals aged 90–100 years who applied to the Internal Medicine Clinic of our hospital in the same period. Exclusion criteria were determined as the presence of known coronary artery disease, acute inflammatory, metabolic diseases, and heart failure for healthy individuals.

Hemogram parameters, glucose, HbA1c, creatinine, and lipid parameters were measured by fasting venous blood sampling at the time of admission in the emergency department or on the first day of hospitalization in the ACS group. For the control group, blood samples collected during outpatient clinic visits were used.

Total cholesterol (TC), low-density lipoprotein (LDL), triglyceride (TG), and high-density lipoprotein (HDL) were obtained by device measurements; the Atherogenic Index of Plasma (AIP) was calculated by the logarithmic transformation of the TG/HDL ratio.

Height, weight, and Body Mass Index (BMI) measurements, other demographic parameters, family history, and smoking habits were obtained retrospectively from the hospital database. Family history was defined as known coronary artery disease or sudden death in at least one first-degree relative, and smoking history was defined as current or past smoking of 20 pack/year or more. Dyslipidemia was defined as abnormal values in at least one lipid parameter: LDL ≥ 130 mg/dL, TG ≥ 150 mg/dL, HDL ≤ 45 mg/dL. Also, definitions such as ’high’ and ’low’ in lipid profile have been clarified to include these specific thresholds. HT was characterized by a systolic blood pressure of 140 mmHg or higher and/or a diastolic pressure of 90 mmHg or above in at least two separate readings, or the ongoing use of antihypertensive medications. Diabetes Mellitus (DM) was defined by elevated blood glucose levels (fasting glucose ≥126 mg/dL, random glucose ≥200 mg/dL, or HbA1c ≥6.5%), confirmed through at least two measurements, or a documented history of antidiabetic treatment. The obesity group included the group with BMI values of 30 and above.

Coronary angiographies were performed with the Judkin technique. Patients who underwent treatments to restore blood flow, such as coronary stenting, balloon angioplasty, or thrombolytic therapy, were included in the revascularization group.

Statistical analysis of the data was performed using SPSS version 29.0 (SPSS Inc., Armonk, NY, USA). Descriptive data were presented as mean ± standard deviation, while categorical variables were reported as percentages. Normally distributed variables between the two groups were analyzed by the independent t-test, and non-normally distributed variables were analyzed by the Mann–Whitney U test. For analyzing differences between categorical variables, the chi square test or Fisher’s Exact-test was used. Traditional risk factors were evaluated by logistic regression analyses. Lipid parameters were converted into categorical (dichotomous) variables and analyzed by the logistic regression method with three different models. Hosmer–Lemeshow fit statistic was used to evaluate the model fit. Multivariate logistic regression analyses were expressed as an odds ratio (OR) with a 95% confidence interval (95% CI). A *p*-value below 0.05 was accepted as a statistically significant difference.

This study was approved by the institutional ethics committee and conducted in accordance with the principles of the Declaration of Helsinki.

## 3. Results

The demographic and biochemical characteristics of the patients with acute coronary syndrome (ACS) and healthy controls included in this study were compared in detail and summarized in Table 1. Significant differences were observed in hemoglobin (Hgb), mean erythrocyte volume (MCV), red cell distribution width (RDW), white blood cell count (WBC), mean platelet volume (MPV), and glucose and total cholesterol values in the ACS group. It was found that Hgb and MCV values were lower and RDW, WBC, MPV, glucose, and total cholesterol values were higher in the ACS group compared to the healthy control group (*p* < 0.05). In lipid profile evaluation, no statistically significant difference was observed between the groups in low-density lipoprotein (LDL) levels (*p* > 0.05). However, high-density lipoprotein (HDL) levels were significantly lower in the ACS group compared to the healthy control group (*p* < 0.001). In addition, the atherogenic index (AIP) was found to be higher in the ACS group compared to the healthy control group (*p* < 0.001). Family history was found to be higher in the healthy control group than in the ACS group and was statistically significant (45.1%, 32.0%, respectively; *p* = 0.049).

In Table 2, patients were classified as NSTEMI and STEMI, and their demographic data, risk factors, biochemical values, and outcomes were compared. Accordingly, it was noticed that among the traditional risk factors, especially, family history was more common in the STEMI group (*p* = 0.049). WBC was significantly higher in the STEMI group (*p* = 0.03). Patients in the STEMI group underwent more CAG and PCI procedures than patients in the NSTEMI group (*p* < 0.001). In-hospital mortality was observed in 7 of 75 patients (9.3%) in the NSTEMI group compared to 13 of 29 patients in the STEMI group, and this difference was statistically significant (*p* < 0.001). No significant difference was observed in the mean length of hospital stay between the two groups.

The effects of gender, family history, smoking history, hypertension, dyslipidemia, DM, and obesity, which are among the traditional risk factors for the development of ACS, were analyzed by logistic regression analyses (Table 3). It was observed that dyslipidemia and obesity increased the risk of ACS development, but this risk was not statistically significant for obesity, whereas a significant level was measured for dyslipidemia (OR = 1.953, 95% CI: 1.034–3.692, *p* = 0.039). Contrary to expectations, the risk of ACS was found to be lower in those with a family history (OR = 0.492, 95% CI: 0.264–0.917, *p* = 0.026).

The effect of lipid parameters on the risk of ACS was evaluated by logistic regression analyses in 3 different models (Table 4). After the variables were classified as high LDL, high TG, low HDL, and high AIP, they were analyzed alone in Model 0, and a statistically significant difference was found only in the low HDL group in this model (OR = 5.534, 95%CI: 2.615–11.713, *p* < 0.001). In Model 1, only the gender variable was added to analyze the existing parameters, and a significant risk was observed only for the low HDL group (OR = 5.619, 95%CI: 2.648–11.921, *p* < 0.001). When the model was adjusted for traditional risk factors such as gender, family history, smoking, hypertension, diabetes, and obesity, in addition to the significant difference in the low HDL group (OR = 5.554, 95% CI: 2.536–12.160, *p* < 0.001), a decreased risk was found for the high LDL group (OR = 0.440, 95% CI: 0.222–0.872, *p* < 0.019). When lipid values were analyzed as continuous variables, a significant risk reduction was observed only for HDL (OR = 0.280, 95% CI: 0.143–0.546, *p* < 0.001), while other lipid parameters, including LDL, TG, and AIP, did not show significant associations. These results emphasize the importance of HDL levels in ACS risk among nonagenarians and highlight the potential protective effect of higher LDL in this age group.

**Figure 1:**
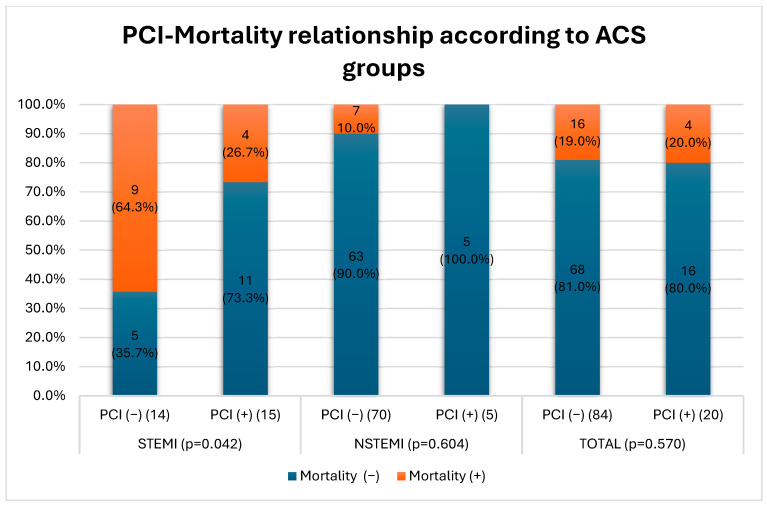
Comparison of mortality rates of patients who underwent PCI (percutaneous coronary intervention) and those who did not according to ACS (acute coronary syndrome) types. ACS, acute coronary syndrome; NSTEMI, non-ST-elevation myocardial infarction; PCI, percutaneous coronary intervention; STEMI, ST-elevation myocardial infarction.

Figure 1 shows the relationship between PCI application and mortality rates in STEMI and NSTEMI groups. In the STEMI group, the mortality rate was 64.3% in patients who did not undergo PCI, whereas the mortality rate was 26.7% in patients who underwent PCI. This difference was statistically significant (*p* = 0.042). In the NSTEMI group, while the mortality rate was 10.0% in patients who did not undergo PCI, no mortality was observed in patients who underwent PCI (0%). However, this difference was not statistically significant (*p* = 0.604). In the total evaluation, the mortality rate was 19.0% in patients who did not undergo PCI and 20.0% in patients who underwent PCI, and again, no significant difference was observed (*p* = 0.570)

**Figure 2:**
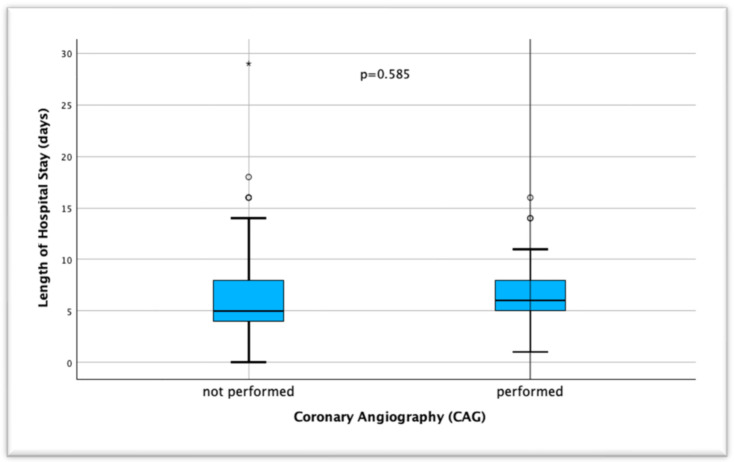
Comparison of the length of hospital stay (days) between patients undergoing and not undergoing coronary angiography (CAG).

When the duration of hospitalization of patients who were interned with a diagnosis of ACS and underwent coronary angiography was compared, the mean duration was 6.68 ± 4.45 (0–29) days in the group without coronary angiography and 6.74 ± 3.74 (1–16) days in the group with angiography, and no significant difference was found between both groups (*p* = 0.585) (Figure 2).

## 4. Discussion

### 4.1. Risk Factors for ACS Development

In individuals over 90 years of age, the effect of classical risk factors on the development of ACS shows a different profile than in younger age groups. In the study conducted by Mostaza et al. in Madrid in 2018, comparing those with and without cardiovascular disease in the population over 90 years of age, male gender, hypertension, dyslipidemia, current smoking, and presence of DM were found to be significantly different as risk factors in the group with cardiovascular disease, and no significant difference was found between BMI values [3]. However, since more than half of the patients in this group were receiving statin therapy, a situation in favor of the cardiovascular disease group was found in atherogenic lipid parameters. Although our study does not include drug use data, it is valuable in terms of showing naive values since patients diagnosed with ACS for the first time were selected. In the GRACE study, which examined the differences in risk factors, treatment and outcomes of 24,165 patients with ACS from 14 different countries according to age groups, it was revealed that factors such as DM, HT, dyslipidemia, and smoking were less common in patients over 85 years of age compared to other age groups. It has been shown that male gender dominance, which reaches 80% in the group under 45 years of age, decreases to 40% over 85 years of age [12]. This suggests that multimorbidity and chronic inflammation processes that increase with age may play a role in the pathogenesis of coronary artery disease rather than traditional risk factors [13,14,15]. To the best of our knowledge, no study to date has compared a substantial cohort of healthy individuals over 90 years old with nonagenarian patients diagnosed with ACS. Therefore, we think that our study may contribute to the literature by comparing patients in the same age group without cardiovascular disease rather than the differences with other age groups. In this context, the fact that the traditional risk factors of male gender, smoking, HT, and DM did not show significant differences in both groups and that BMI, HbA1c, and creatinine values were similar clearly reveals the etiological differences in this age group [16,17]. In our study, we observed that dyslipidemia significantly increased the risk of ACS, and this finding is consistent with the literature emphasizing the importance of lipid control in the elderly [17,18]. Although the study by Krumholz et al. in 1994 showed that dyslipidemia was not associated with cardiovascular risk and mortality in patients over 70 years of age, our study clearly demonstrates that especially low HDL alone constitutes a risk [19]. Other than that, the finding that high LDL levels may reduce the risk of ACS in elderly patients is not entirely unexpected, as a comprehensive review by Ravnskov et al. in 2016 also reported similar results [20]. However, since data on patients’ statin use are not available, the predictive value of this finding in terms of lipid-lowering therapy remains debatable. Additionally, although a decreased risk was observed in the high LDL group, this reduction in risk was not observed when LDL was analyzed as a continuous variable per 10 mg/dL increase, suggesting that the protective effect may be influenced by categorization rather than a dose-response relationship. The lower prevalence of the family history of CAD in the ACS group compared to healthy nonagenarians raises intriguing questions. While competing risks may explain early mortality in individuals with genetic predisposition, it remains noteworthy that those with a family history survived within the healthy cohort [21].

In our findings, hemoglobin and MCV values were found to be low, whereas parameters such as RDW, WBC, glucose, and total cholesterol were found to be high in the ACS group. Although this difference was statistically significant when glucose values in the ACS group were analyzed in the emergency department, regardless of adequate fasting time, this limitation was not considered to have clinical significance in the current situation since no significant difference was observed, especially in comparisons related to HbA1c levels or the presence of DM. Especially high RDW and WBC levels have been found to be higher in the elderly population with cardiovascular disease as a result of inflammation in the literature, and its association with mortality has been shown in the presence of ACS [22,23,24].

### 4.2. Differences According to ACS Groups

When the patients were grouped as NSTEMI and STEMI, only family history showed a significant difference in terms of risk factors. Yayan et al. showed that only hypertension was statistically higher in the NSTEMI group between NSTEMI and STEMI groups in individuals over 90 years of age, but family history was not among the traditional risk factors [25]. In this sense, our study is valuable in terms of emphasizing the importance of family history in ACS type. Although the limited sample size may restrict definitive conclusions, the potential role of genetic factors in promoting plaque instability and more severe presentations, such as STEMI, should not be overlooked, as family history may contribute to a more atherogenic environment, leading to critical coronary lesions. In addition to the limited sample size, the absence of coronary imaging for all patients prevents a comprehensive assessment of plaque characteristics and burden.

We think that the higher leukocyte values in the STEMI group are related to the severity of the inflammation. Di Stefano et al. showed that inflammatory markers such as leukocytes, high-sensitive C-reactive protein (hs-CRP), ferritin, and interleukin-6 (IL-6) were much higher in STEMI patients than in the NSTEMI group in their study in middle-aged patients [26]. One of the limitations of our study is that inflammatory markers other than leukocytes were not included, but the differences in leukocyte values support the existing literature.

Sugiyama et al., in their study including patients over 30 years of age between 2001 and 2011, when they analyzed PCI intervention rates according to ACS type over the years, showed that the PCI rate was 76.6% in the STEMI group and 33.9% in the NSTEMI group by 2011 [27]. Although our study shows similarly high rates for the STEMI group, with a PCI rate of up to 6.7% in the NSTEMI group, it reveals a much more conservative trend in this group.

Mortality rates in the STEMI group were significantly higher than in the NSTEMI group (44.8% and 9.3%, respectively). Sheldon et al. also showed that mortality was higher in the STEMI group than in the NSTEMI group and that this group especially benefited more from PCI interventions in their study, including patients over 90 years of age [28]. It was thought that the differences in mortality rates between both groups may be effective in the tendency of clinicians towards PCI.

### 4.3. Invasive Treatment and Mortality Reduction

In their 2008 study, From et al. showed that in-hospital mortality after PCI intervention decreased from 22% to 6% in patients over 90 years of age compared to the pre-2000s and argued that PCI should not be avoided in advanced age groups if indicated due to the increased technical success of the procedure [29]. In our study, CAG was performed in 8 (10.7%) and PCI in 5 (6.7%) of 75 patients in the NSTEMI group, whereas in the STEMI group, CAG was performed in 19 (65.5%) and PCI in 15 (51.1%) of 29 patients (*p* < 0.001) (Table 2). In our study, the decision not to perform coronary angiography in many nonagenarian patients was primarily influenced by advanced age, frailty, multiple comorbidities, and patient or family preferences. Like the SENIOR-RITA trial, which demonstrated no significant difference in cardiovascular outcomes between invasive and conservative strategies in older adults with NSTEMI, our findings highlight the complex decision-making process in this population [30]. While SENIOR-RITA focused on patients ≥75 years, our cohort of nonagenarians represents an even more vulnerable group, where the risks associated with invasive procedures may outweigh potential benefits. Several studies have shown that compared with younger patient groups, patients older than 90 years of age have higher short- and long-term mortality rates after PCI [31,32,33]. Some studies comparing ACS patients over 90 years of age with PCI and medical therapy in their own age groups have shown that in-hospital or 1-year mortality rates, MACE, and all-cause mortality were lower in the PCI group [34,35,36]. In our study, no significant difference was found when the relationship between PCI application and mortality in all ACS patients was analyzed (*p* = 0.570), but when classified according to ACS subgroups, higher in-hospital mortality was observed in patients who underwent PCI, especially in the STEMI group, compared to the medical treatment arm (*p* = 0.042) (Figure 1). Şahin et al. and Cepas-Guillen et al. showed lower mortality rates for the STEMI group, which is similar to our study [9,37]. In addition, the fact that no statistically significant difference was observed between patients without and with CAG in terms of the mean duration of hospitalization may be a reason not to avoid PCI. From et al. defined a shorter hospitalization duration of 3.7 days in patients of the same age group, which is shorter than the mean duration in our study [29]. However, we believe that especially in vulnerable patient groups with high comorbidity, there may be differences in the duration of hospitalization at the clinician’s initiative to observe post-procedural complications, if any.

### 4.4. Limitations and Significance of This Study

In this study, the demographic, biochemical, and lipid profiles of patients over 90 years of age with acute coronary syndrome (ACS) and healthy controls were compared in detail, and the relationship between different types of ACS and risk factors was evaluated. Our findings demonstrated once again the importance of traditional risk factors in the development of ACS in the older age group, with low HDL levels being a particularly prominent risk factor. Furthermore, we observed that invasive treatment approaches were effective in reducing mortality in the STEMI group, and family history played a greater role in this group compared to the NSTEMI group. As a contribution to the literature, our results emphasize the importance of personalized treatment approaches in this age group. Nonetheless, our study has certain limitations, primarily due to its retrospective design. One major limitation of this study is the different timing of blood sampling: blood samples for ACS patients were collected during an acute event, while samples for the control cohort were obtained during elective presentations. This discrepancy might introduce a significant bias, particularly affecting inflammatory markers or glucose. However, lipid profiles or HbA1c values were obtained under appropriate conditions after hospitalization rather than during the acute admission, which supports their reliability. This methodological limitation should be considered when interpreting the biochemical results. The lack of retrospective medical treatment history of the patients, the fact that the control group was selected among individuals who were admitted to the hospital and had a possible health problem, the lack of some biochemical markers (such as inflammatory markers, lipoprotein a, homocysteine, etc.) limit the generalizability of the results. Our study is also limited by the absence of detailed data on periprocedural complications and post-discharge outcomes, which restricts a comprehensive evaluation of the safety and long-term impact of invasive strategies in nonagenarians. Additionally, although comorbidities as traditional risk factors were assessed, the lack of an objective frailty assessment represents a significant limitation. Frailty is increasingly recognized as a critical factor in clinical decision-making for elderly patients, and its absence in our analysis may affect the generalizability and applicability of our findings to broader elderly populations, as highlighted by the SENIOR-RITA trial and current clinical guidelines [30]. In future studies, prospective studies should aim to contribute to the clinical management strategies of ACS patients in this age group.

## 5. Conclusions

In conclusion, this study reveals that low HDL levels are a significant risk factor for the development of acute coronary syndrome (ACS) in individuals over 90 years of age, aligning with existing literature on lipid control in the elderly. However, it also highlights critical differences within the ACS group itself, emphasizing the distinct profiles of STEMI and NSTEMI patients. Our findings demonstrate that family history plays a more prominent role in STEMI cases compared to NSTEMI, suggesting genetic predispositions might influence the severity of myocardial infarction in this age group. Additionally, PCI interventions showed significant benefits in reducing in-hospital mortality rates in STEMI patients, reinforcing the importance of timely and appropriate invasive treatments, even in advanced age groups.

Although PCI was not associated with significant mortality differences in the NSTEMI group, it remains a valuable therapeutic option that may improve long-term outcomes. These results suggest that ACS patients in nonagenarian populations require personalized treatment approaches that consider both their unique risk profiles and the potential benefits of invasive strategies. Clinicians should carefully assess the balance between potential procedural risks and the expected benefits, particularly in STEMI patients, who appear to benefit more substantially from PCI.

This study underscores the importance of tailoring cardiovascular care in the elderly to account for age-specific differences in disease presentation and treatment response. Future studies should emphasize prospective designs to better assess the long-term outcomes of PCI in nonagenarian patients with ACS and refine guidelines for invasive procedures in this growing demographic.

## Figures and Tables

**Table 1:** Comparison of the demographic and biochemical characteristics of the healthy control group and the acute coronary syndrome group.

	CONTROL	ACS	*p*
***n***	113 (52.3)	104 (47.7)	
**Age**	91 ± 2.2	91.3 ± 1.8	0.302
**Male**	40 (35.4)	38 (36.5)	0.861
**BMI**	26.6 ± 3.3	26.9 ± 4.8	0.896
**Smoking**	35 (31.0)	38 (36.9)	0.358
**HT**	91 (80.5)	72 (69.9)	0.070
**DM**	27 (23.9)	25 (24.0)	0.980
**Family history**	51 (45.1)	33 (32.0)	**0.049**
**Biochemical Parameters**			
**Hgb(g/dL)**	12.4 ± 1.4	11.8 ± 1.8	**0.008**
**MCV (fL)**	89.6 ± 6.9	87.4 ± 6.8	**0.009**
**RDW (%)**	15.1 ± 1.9	15.6 ± 2.0	**0.020**
**WBC (10^3^/µL)**	7.2 ± 1.7	10.1 ± 3.7	**<0.001**
**PLT (10^3^/µL)**	229.2 ± 77.9	232.5 ± 82.4	0.902
**MPV (fL)**	9.4 ± 1.9	8.7 ± 1.5	**0.021**
**Glucose (mg/dL)**	111.3 ± 32.0	152.9 ± 71.7	**<0.001**
**HbA1c (%)**	6.23 ± 0.9	6.29 ± 1.01	0.910
**Creatinine (mg/dL)**	1.09 ± 0.3	1.16 ± 0.52	0.517
**Lipid Profiles**			
**TC (mg/dL)**	197.9 ± 44.1	179.6 ± 42.7	**0.003**
**LDL (mg/dL)**	119.7 ±36.9	115.0 ± 36.2	0.272
**TG (mg/dL)**	114.6 ± 47.7	123.4 ± 47.7	0.612
**HDL (mg/dL)**	55.5 ± 15.9	38.6 ± 12.1	**<0.001**
**AIP**	0.299 ± 0.24	0.485 ± 0.264	**<0.001**

ACS, acute coronary syndrome; AIP, atherogenic index of plasma; BMI, body mass index; DM, diabetes mellitus; HDL, high density lipoprotein; Hgb, hemoglobin; HT, hypertension; LDL, low density lipoprotein; MCV, mean corpuscular volume; MPV, mean platelet volume; PLT, platelet; RDW, red-cell distribution width; TC, total cholesterol; TG, triglyceride; WBC, white blood cell.

**Table 2:** Comparison of the demographic and biochemical data of patients with acute coronary syndrome according to MI type.

	NSTEMI	STEMI	*p*
***n***	75 (72.1)	29 (27.9)	
**Age**	91.3 ± 2.1	90.8 ± 1.40	0.442
**Male**	24 (32)	14 (48.3)	0.122
**BMI**	27.1 ± 5.6	27.4 ± 3.1	0.865
**Smoking**	26 (35.1)	12 (41.4)	0.555
**HT**	55 (74.3)	17 (58.6)	0.118
**DM**	21 (28.0)	4 (13.8)	0.128
**Family history**	15 (20.3)	18 (62.1)	**<0.001**
**Biochemical Parameters**			
**Hgb**	11.9 ± 1.8	11.2 ± 1.8	0.142
**MCV (fL)**	86.6 ± 6.7	87.7 ± 8.3	0.893
**RDW (%)**	15.4 ± 1.8	16.1 ± 2.8	0.324
**WBC (10^3^/µL)**	9.78 ± 3.70	11.48 ± 4.20	**0.030**
**PLT (10^3^/µL)**	233.5 ± 90.3	231.2 ± 59.1	0.798
**MPV (fL)**	8.93 ± 1.60	8.31 ± 1.03	0.721
**Glucose (mg/dL)**	152.5 ± 73.9	145.4 ± 67.5	0.364
**HbA1c (%)**	6.40 ± 0.99	6.15 ± 1.14	0.422
**Creatinine (mg/dL)**	1.12 ± 0.52	1.18 ± 0.48	0.522
**Lipid Profiles**			
**TC (mg/dL)**	177.4 ± 38.7	187.3 ± 54.9	0.461
**LDL (mg/dL)**	112.2 ± 33.4	124.9 ± 44.4	0.167
**TG (mg/dL)**	127.7 ± 70.7	108.0 ± 41.9	0.285
**HDL (mg/dL)**	39.1 ± 12.5	36.9 ± 10.9	0.426
**AIP**	0.486 ± 0.284	0.460 ± 0.182	0.627
**CAG**	8 (10.7)	19 (65.5)	**<0.001**
**PCI**	5 (6.7)	15 (51.7)	**<0.001**
**Length of hospital stay (days)**	5.7 ± 4.3	6.6 ± 4.1	0.781
**Mortality**	7 (9.3)	13 (44.8)	**<0.001**

AIP, atherogenic index of plasma; BMI, body mass index; CAG, coronary angiography; DM, diabetes mellitus; HDL, high density lipoprotein; Hgb, hemoglobin; HT, hypertension; LDL, low density lipoprotein; MCV, mean corpuscular volume; MPV, mean platelet volume; NSTEMI, non-ST-elevation myocardial infarction; PCI, percutaneous coronary intervention; PLT, platelet; RDW, red-cell distribution width; STEMI, ST-elevation myocardial infarction; TC, total cholesterol; TG, triglyceride; WBC, white blood cell.

**Table 3:** Effects of traditional risk factors on the likelihood of developing acute coronary syndrome.

	ACS	
	95% CI	*p*
**Sex**	0.905 (0.482–1.699)	0.755
**Family history**	0.492 (0.264–0.917)	**0.026**
**Smoking**	1.868 (0.955–3.655)	0.068
**HT**	0.525 (0.268–1.027)	0.060
**DM**	1.038 (0.533–2.024)	0.912
**Dyslipidemia**	1.953 (1.034–3.692)	**0.039**
**Obesity**	1.777 (0.856–3.687)	0.123

ACS, acute coronary syndrome; DM, diabetes mellitus; HT, hypertension.

**Table 4:** Acute coronary syndrome risks according to lipid profiles.

	Crude OR	Adjusted OR	
	Model 0 OR (95% CI) *p*-Value	Model 1 OR (95% CI) *p*-Value	Model 2 OR (95% CI) *p*-Value
**High LDL**	0.544 (0.287–1.033) 0.063	0.528 (0.277–1.009) 0.053	0.440 (0.222–0.872) **0.019**
**LDL** (per 10 mg/dL increase)	1.023 (0.926–1.130) 0.656	1.016 (0.918–1.123) 0.765	0.997 (0.897–1.110) 0.963
**High TG**	1.054 (0.460–2.416) 0.901	0.980 (0.416–2.307) 0.962	1.047 (0.424–2.585) 0.921
**TG** (per 10 mg/dL increase)	1.046 (0.888–1.231) 0.594	1.036 (0.881–1.217) 0.669	1.027 (0.860–1.226) 0.770
**Low HDL**	5.534 (2.615–11.713) **<0.001**	5.619 (2.648–11.921) **<0.001**	5.554 (2.536–12.160) **<001**
**HDL** (per 10 mg/dL increase)	0.280 (0.143–0.546) **< 0.001**	0.271 (0.139–0.528) < 0.001	0.274 (0.134–0.562) **< 0.001**
**High AIP**	0.933 (0.406–2.142) 0.869	0.955 (0.415–2.202) 0.915	1.061 (0.439–2.564) 0.895
**AIP** (per 1 unit increase)	0.055 (0.000–12.083) 0.292	0.054 (0.000–11.218) 0.204	0.078 (0.000–25.293) 0.387

AIP, atherogenic index of plasma; CI, confidence interval; HDL, high-density lipoprotein; LDL, low-density lipoprotein; OR, odds ratio; TG, triglyceride. Model 0: unadjusted *χ*^2^ = 39.3; df = 4; *p* < 0.001; log likelihood = 261.0; Cox and Snell = 0.166; *R^2^* Nagelkerke = 0.221. Model 1: adjusted for sex *χ*^2^ = 39.8; df = 5; *p* < 0.001; log likelihood = 260.5; Cox and Snell = 0.168; *R^2^* Nagelkerke = 0.224. Model 2: adjusted for sex, smoking, family history, hypertension, diabetes, and obesity *χ*^2^ = 52.4; df = 10; *p* < 0.001; log likelihood = 246.5; Cox and Snell = 0.216; *R^2^* Nagelkerke = 0.288.

## Data Availability

Research data are not publicly available but can be shared by the corresponding author upon request.

## References

[1] United Nations (2024). Population Division, Department of Economic and Social Affairs. World Population Prospects 2024.

[2] Leucker T.M., Gerstenblith G. (2023). Cardiovascular Disease in the Elderly.

[3] Mostaza J.M., Lahoz C., Salinero-Fort M.A., Cardenas J. (2019). Cardiovascular Disease in Nonagenarians: Prevalence and Utilization of Preventive Therapies. Eur. J. Prev. Cardiolog..

[4] Tsao C.W., Aday A.W., Almarzooq Z.I., Anderson C.A.M., Arora P., Avery C.L., Baker-Smith C.M., Beaton A.Z., Boehme A.K., Buxton A.E. (2023). Heart Disease and Stroke Statistics—2023 Update: A Report From the American Heart Association. Circulation.

[5] Rich M.W. (2006). Epidemiology, Clinical Features, and Prognosis of Acute Myocardial Infarction in the Elderly. Am. J. Geri. Cardiol..

[6] Forman D.E., Maurer M.S., Boyd C., Brindis R., Salive M.E., Horne F.M., Bell S.P., Fulmer T., Reuben D.B., Zieman S. (2018). Multimorbidity in Older Adults with Cardiovascular Disease. J. Am. Coll. Cardiol..

[7] Couture E.L., Farand P., Nguyen M., Allard C., Wells G.A., Mansour S., Rinfret S., Afilalo J., Eisenberg M., Montigny M. (2018). Impact of an Invasive Strategy in the Elderly Hospitalized with Acute Coronary Syndrome with Emphasis on the Nonagenarians. Catheter. Cardiovasc. Interv..

[8] Shah P., Najafi A.H., Panza J.A., Cooper H.A. (2009). Outcomes and Quality of Life in Patients ≥85 Years of Age With ST-Elevation Myocardial Infarction. Am. J. Cardiol..

[9] Cepas-Guillén P.L., Echarte-Morales J., Caldentey G., Gómez E.M., Flores-Umanzor E., Borrego-Rodriguez J., Llagostera M., Viana Tejedor A., Vidal P., Benito-Gonzalez T. (2022). Outcomes of Nonagenarians with Acute Coronary Syndrome. J. Am. Med. Dir. Assoc..

[10] Goel K., Gupta T., Gulati R., Bell M.R., Kolte D., Khera S., Bhatt D.L., Rihal C.S., Holmes D.R. (2018). Temporal Trends and Outcomes of Percutaneous Coronary Interventions in Nonagenarians. JACC Cardiovasc. Interv..

[11] Jokhadar M., Wenger N.K. (2009). Review of the Treatment of Acute Coronary Syndrome in Elderly Patients. Clin. Interv. Aging.

[12] Zimmerman F.H., Cameron A., Fisher L.D., Grace N. (1995). Myocardial Infarction in Young Adults: Angiographic Characterization, Risk Factors and Prognosis (Coronary Artery Surgery Study Registry). J. Am. Coll. Cardiol..

[13] Ferrucci L., Fabbri E. (2018). Inflammageing: Chronic Inflammation in Ageing, Cardiovascular Disease, and Frailty. Nat. Rev. Cardiol..

[14] Liberale L., Montecucco F., Tardif J.-C., Libby P., Camici G.G. (2020). Inflamm-Ageing: The Role of Inflammation in Age-Dependent Cardiovascular Disease. Eur. Heart J..

[15] García-Blas S., Cordero A., Diez-Villanueva P., Martinez-Avial M., Ayesta A., Ariza-Solé A., Mateus-Porta G., Martínez-Sellés M., Escribano D., Gabaldon-Perez A. (2021). Acute Coronary Syndrome in the Older Patient. J. Clin. Med..

[16] Kannel W.B. (1961). Factors of Risk in the Development of Coronary Heart Disease—Six-Year Follow-up Experience: The Framingham Study. Ann. Intern. Med..

[17] Kannel W.B. (2002). Coronary Heart Disease Risk Factors in the Elderly. Am. J. Geriatr. Cardiol..

[18] Wenger N.K. (2004). Dyslipidemia as a Risk Factor at Elderly Age. Am. J. Geriatr. Cardiol..

[19] Krumholz H.M., Seeman T.E., Merrill S.S., de Leon C.F.M., Vaccarino V., Silverman D.I., Tsukahara R., Ostfeld A.M., Berkman L.F. (1994). Lack of Association between Cholesterol and Coronary Heart Disease Mortality and Morbidity and All-Cause Mortality in Persons Older than 70 Years. JAMA.

[20] Ravnskov U., Diamond D.M., Hama R., Hamazaki T., Hammarskjöld B., Hynes N., Kendrick M., Langsjoen P.H., Malhotra A., Mascitelli L. (2016). Lack of an Association or an Inverse Association between Low-Density-Lipoprotein Cholesterol and Mortality in the Elderly: A Systematic Review. BMJ Open.

[21] Madhavan M.V., Gersh B.J., Alexander K.P., Granger C.B., Stone G.W. (2018). Coronary Artery Disease in Patients ≥ 80 Years of Age. J. Am. Coll. Cardiol..

[22] Liu X.-M., Ma C.-S., Liu X.-H., Du X., Kang J.-P., Zhang Y., Wu J.-H. (2015). Relationship between Red Blood Cell Distribution Width and Intermediate-Term Mortality in Elderly Patients after Percutaneous Coronary Intervention. J. Geriatr. Cardiol..

[23] Xanthopoulos A., Tryposkiadis K., Dimos A., Bourazana A., Zagouras A., Iakovis N., Papamichalis M., Giamouzis G., Vassilopoulos G., Skoularigis J. (2021). Red Blood Cell Distribution Width in Elderly Hospitalized Patients with Cardiovascular Disease. World J. Cardiol..

[24] Weijenberg M.P., Feskens E.J.M., Kromhout D. (1996). White Blood Cell Count and the Risk of Coronary Heart Disease and All-Cause Mortality in Elderly Men. Arterioscler. Thromb. Vasc. Biol..

[25] Yayan J. (2014). Association of Traditional Risk Factors with Coronary Artery Disease in Nonagenarians: The Primary Role of Hypertension. Clin. Interv. Aging.

[26] Di Stefano R., Di Bello V., Barsotti M.C., Grigoratos C., Armani C., Dell’Omodarme M., Carpi A., Balbarini A. (2009). Inflammatory Markers and Cardiac Function in Acute Coronary Syndrome: Difference in ST-Segment Elevation Myocardial Infarction (STEMI) and in Non-STEMI Models. Biomed. Pharmacother..

[27] Sugiyama T., Hasegawa K., Kobayashi Y., Takahashi O., Fukui T., Tsugawa Y. (2015). Differential Time Trends of Outcomes and Costs of Care for Acute Myocardial Infarction Hospitalizations by ST Elevation and Type of Intervention in the United States, 2001–2011. J. Am. Heart Assoc..

[28] Sheldon M., Blankenship J.C. (2022). STEMI in Nonagenarians: Never Too Old. Catheter. Cardiovasc. Interv..

[29] From A.M., Rihal C.S., Lennon R.J., Holmes D.R., Prasad A. (2008). Temporal Trends and Improved Outcomes of Percutaneous Coronary Revascularization in Nonagenarians. JACC Cardiovasc. Interv..

[30] Kunadian V., Mossop H., Shields C., Bardgett M., Watts P., Teare M.D., Pritchard J., Adams-Hall J., Runnett C., Ripley D.P. (2024). Invasive Treatment Strategy for Older Patients with Myocardial Infarction. N. Engl. J. Med..

[31] Sawant A.C., Josey K., Plomondon M.E., Maddox T.M., Bhardwaj A., Singh V., Rajagopalan B., Said Z., Bhatt D.L., Corbelli J. (2017). Temporal Trends, Complications, and Predictors of Outcomes Among Nonagenarians Undergoing Percutaneous Coronary Intervention. JACC Cardiovasc. Interv..

[32] Tokarek T., Siudak Z., Dziewierz A., Rakowski T., Krycińska R., Siwiec A., Dudek D. (2018). Clinical Outcomes in Nonagenarians Undergoing a Percutaneous Coronary Intervention: Data from the ORPKI Polish National Registry 2014–2016. Coron. Artery Dis..

[33] Antonsen L., Jensen L.O., Terkelsen C.J., Tilsted H., Junker A., Maeng M., Hansen K.N., Lassen J.F., Thuesen L., Thayssen P. (2013). Outcomes after Primary Percutaneous Coronary Intervention in Octogenarians and Nonagenarians with ST-segment Elevation Myocardial Infarction: From the Western Denmark Heart Registry. Catheter. Cardiovasc. Interv..

[34] Oh S., Jeong M.H., Cho K.H., Kim M.C., Sim D.S., Hong Y.J., Kim J.H., Ahn Y. (2022). Outcomes of Nonagenarians with Acute Myocardial Infarction with or without Coronary Intervention. J. Clin. Med..

[35] Lee K.H., Ahn Y., Kim S.S., Rhew S.H., Jeong Y.W., Jang S.Y., Cho J.Y., Jeong H.C., Park K.-H., Yoon N.S. (2014). Characteristics, In-Hospital and Long-Term Clinical Outcomes of Nonagenarian Compared with Octogenarian Acute Myocardial Infarction Patients. J. Korean Med. Sci..

[36] Wu Y.-J., Hou C.J.-Y., Chou Y.-S., Tsai C.-H. (2004). Percutaneous Coronary Intervention in Nonagenarians. Acta Cardiol. Sin..

[37] Sahin M., Ocal L., Kalkan A.K., Kilicgedik A., Kalkan M.E., Teymen B., Arslantas U., Turkmen M.M. (2017). In-Hospital and Long Term Results of Primary Angioplasty and Medical Therapy in Nonagenarian Patients with Acute Myocardial Infarction. J. Cardiovasc. Thorac. Res..

